# Superior oblique paresis after endoscopic brow lift: A case report

**DOI:** 10.1002/ccr3.9044

**Published:** 2024-07-03

**Authors:** Farid Shekarchian, Mehrdad Motamed Shariati, Mitra Karimi Amir Abadi

**Affiliations:** ^1^ Eye Research Center Mashhad University of Medical Sciences Mashhad Iran

**Keywords:** diplopia, forehead lift procedure, infrared reflectance image, superior oblique paresis

## Abstract

**Key Clinical Message:**

Although a forehead lift is generally a safe surgery, it has well‐known complications. Iatrogenic SO paresis is one of the rare complications following forehead lifting procedures which almost resolves spontaneously.

**Abstract:**

This report aims to introduce a woman with superior oblique (SO) muscle paresis following the brow and forehead lift procedure. A 30‐year‐old woman with a history of brow and forehead lift surgery was referred to the ophthalmic emergency department complaining of vertical diplopia. A right eye hypertropia was obvious at the left gaze. A Park's three‐step test showed right eye superior oblique paresis. Other ophthalmic examinations including slit‐lamp examination, tonometry, and dilated fundoscopy were unremarkable for both eyes. After a 3‐month follow‐up period, she had no diplopia. No sign of SO paresis was apparent in her ocular motility examinations. Iatrogenic SO paresis is one of the rare complications following forehead lifting procedures which almost resolves spontaneously.

## 1 INTRODUCTION

The brow and forehead lift is a common cosmetic surgery used for facial rejuvenation. It has gained popularity in recent years.^1^, ^2^ Due to the growing trend toward brow lift procedures, it is prudent to recognize, prevent, and manage its complications, as they can be distressing for both patient and surgeon. Endoscopic brow and forehead lift is a surgical technique for forehead rejuvenation that is performed through small 1‐cm incisions behind to site of the hairline. After the skin incision, endoscopically the tissue dissects from the periosteum and the forehead fixes to the periosteum in various ways. This surgical technique through small incisions causes faster postoperative recovery time. Although new advanced techniques have been developed to improve aesthetic results, the main complications include hematoma, alopecia, scarring formation, and motor and sensory nerve injuries.^3^ Because of the proximity of the trochlea in the supranasal area to the surgical site and tissue dissection site, trochlea and superior oblique tendon injury is possible by tissue dissection. Major risk factors contributing to a higher incidence of complications are old age, male gender, hypertension, smoking, and the use of anticoagulants, antiplatelet, and nonsteroidal anti‐inflammatory drugs.^4^

This report aims to introduce a woman with superior oblique (SO) muscle paresis following the brow and forehead lift procedure. Also, we showed the value of the posterior pole (P.pole) infrared reflectance image to measure the abnormal ocular torsion quantitatively.

## 2 CASE HISTORY

**FIGURE 1:**
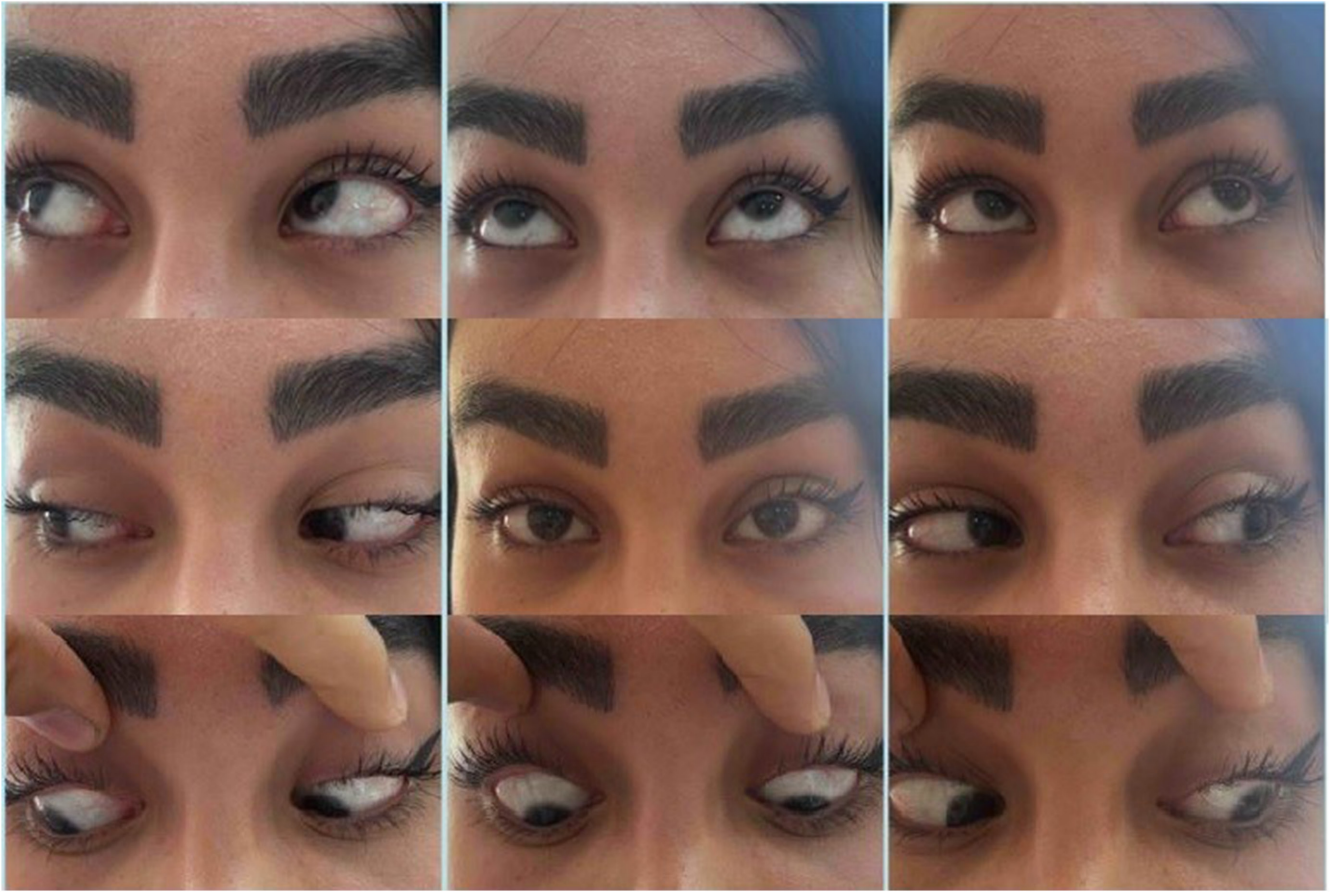
Nine gaze of reported patient 2 weeks after endoscopic forehead lift shows right hypertropia that increases in left gaze and right head tilt.

**FIGURE 2:**
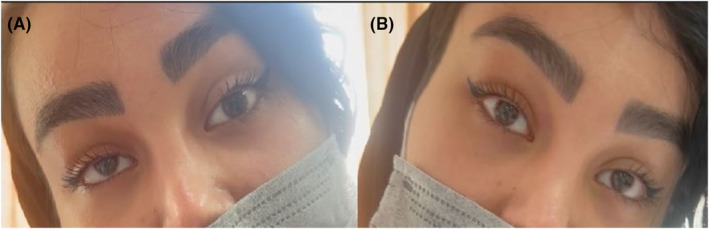
(A) Right head tilt with right hypertropia. (B) Left head tilt shows no tropia.

**FIGURE 3:**
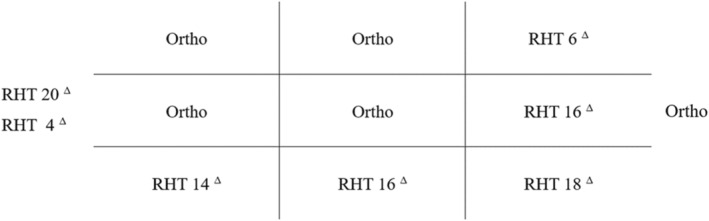
The deviometery shows right hypertropia that increases in left and down gaze. RHT, right hypertropia.

A 30‐year‐old woman with no specific medical history was referred to the ophthalmic emergency department complaining of vertical diplopia. The patient's symptoms started 2 weeks earlier immediately after a brow and forehead lift surgery. The eyes were orthotropic at the primary position. A right eye hypertropia was obvious at the left gaze (Figures 1 and 2). A Park's three‐step test showed right eye superior oblique paresis (Figure 3). Other ophthalmic examinations including slit‐lamp examination, tonometry, and dilated fundoscopy were unremarkable for both eyes.

## 3 METHODS (INVESTIGATIONS AND TREATMENT)

**FIGURE 4:**
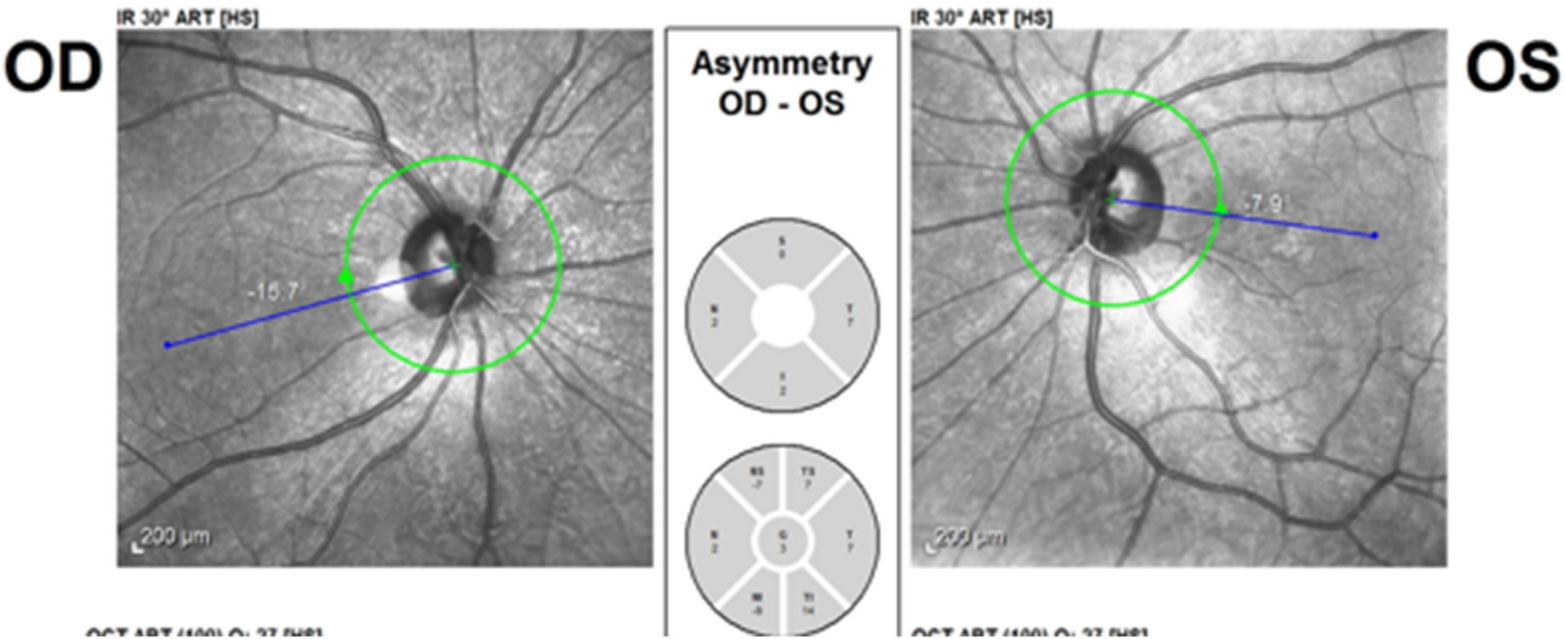
Fifteen degrees of extorsion of the right eye after forehead lift surgery.

The P.pole infrared reflectance image (Heidelberg Eye Explorer version 1.9.13.0, Spectralis Viewing Module 6.5.2.0; Heidelberg Engineering) showed right eye extorsion with a fovea to disc (FoDi) axis angle of −15.7° (Figure 4). Considering the normal upper limit of −12° for the FoDi‐axis, SO paresis was confirmed. The patient was followed with the diagnosis of iatrogenic SO paresis.

## 4 CONCLUSIONS AND RESULTS

**FIGURE 5:**
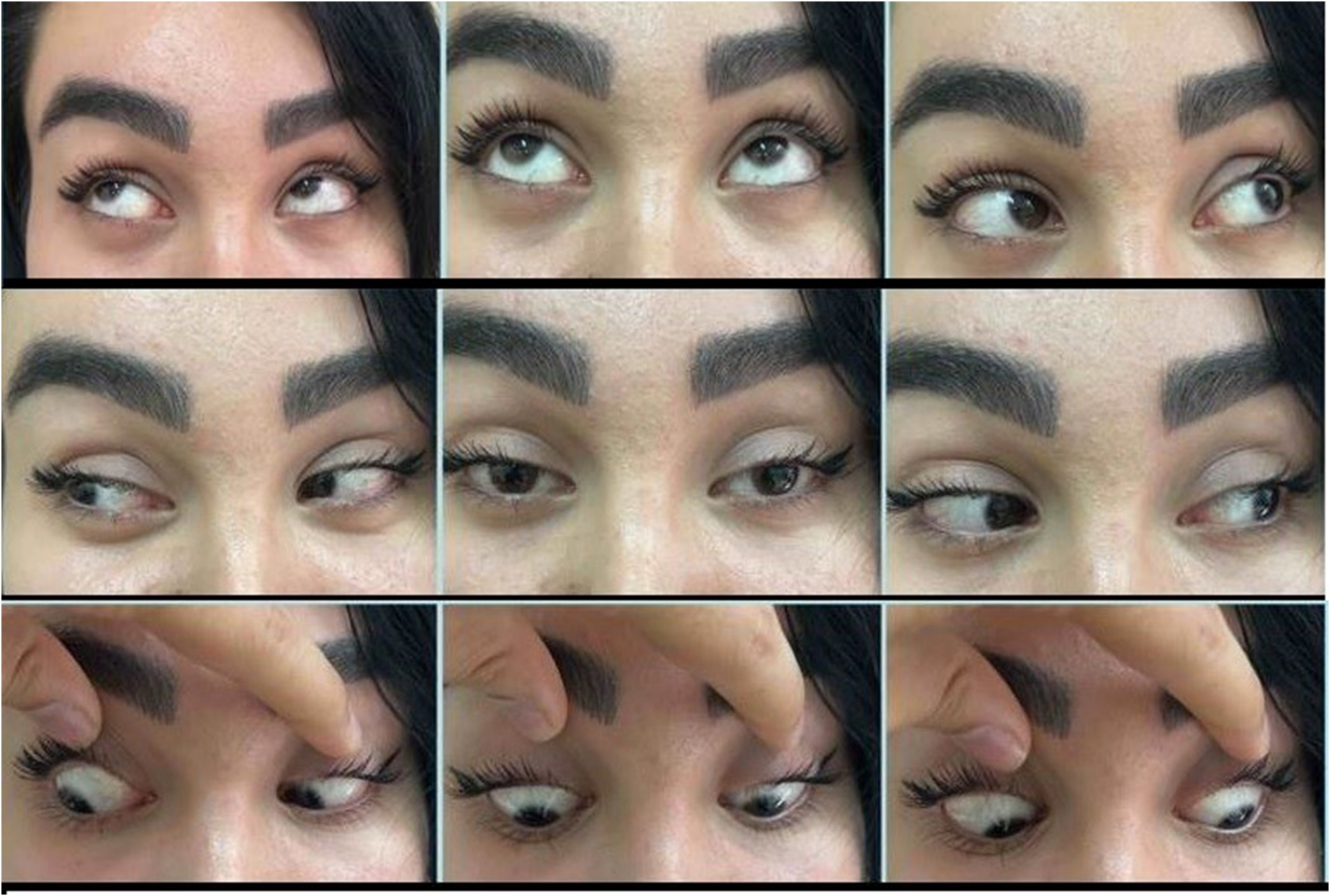
The nine gaze of the reported patient 3 months after the endoscopic forehead lift shows resolution of the right hypertropia.

**FIGURE 6:**
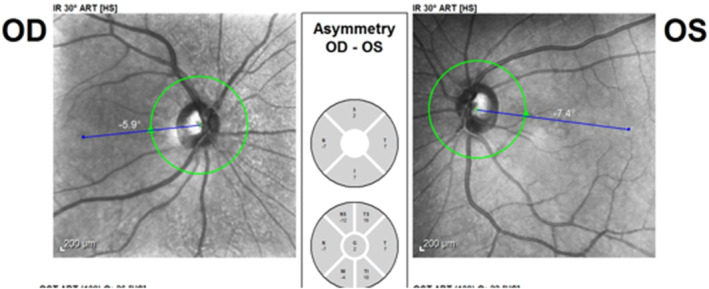
Recovery of the right eye extorsion 3 months after forehead lift surgery.

After a 3‐month follow‐up period, she had no diplopia. No sign of SO paresis was apparent in her ocular motility examinations and the P.pole infrared reflectance image (Figures 5 and 6).

## 5 DISCUSSION

The brow and forehead lift procedure is generally considered safe; however, some complications are well established. Generally, there are two categories of brow elevation and forehead lifts: nonsurgical and surgical methods. Examples of nonsurgical methods include botulinum toxin A injection and soft tissue fillers which have temporary results and necessitate less experience compared to surgical procedures. Surgical techniques can be classified into three main categories: trans‐forehead eyebrow/forehead lift, trans‐blepharoplasty eyebrow lift, and direct eyebrow lift.^5^ Although there are numerous forehead lift techniques, none of them has been proven to be superior in terms of lower postoperative complication rate. The risk of complications can be predicted and minimized through perioperative patient evaluation, mastery of the anatomy of the region, and intraoperative modifications, and planning.^6^ There have been different complications following brow lifts, such as numbness, alopecia, hematoma, flap necrosis, infection, lagophthalmos, and palpable sutures, most of which are non‐life‐threatening and are managed conservatively,^7^ as in our case where superior oblique paresis has been resolved spontaneously within 3 months of follow‐up.

In the case of superior oblique paresis, patients complain of vertical or torsional diplopia. The diplopia is binocular and incomitant. For example, in right superior oblique involvement, the maximum vertical diplopia is observed when the right eye is directed downward and inward. Clinical evaluation of patients with superior oblique paresis shows abnormal head posture (torticollis) and strabismus.^8^ We used the P.pole infrared reflectance image to measure the amount of cyclo deviation. The idea of calculating the amount of rotational deviation from fundus photographs was investigated previously.^9^, ^10^, ^11^ According to the patient's first examination, and the result of the FoDi axis angle measurement which was 15.7 degrees of extorsion (maximum normal extorsion is 12 degrees), the patient was diagnosed as right superior oblique iatrogenic paresis following brow lift, which is an uncommon complication. Although the exact incidence and mechanism are not well understood, according to a study by Shim et al. it is estimated to be 1%.^12^ We can suggest different mechanisms for SO paresis in this patient including traumatic trochlear injury, damage to SO muscle due to local anesthetic toxicity or hematoma, injuries during cauterizing and clamping, and localized postoperative edema. Considering the patient's expectation of lifting the medial part of the eyebrow in addition to the lateral part, the possibility of trauma to the trochlea during surgery increases. Besides, the presence of hypoesthesia in the trochlear region supports the hypothesis of traumatic injury. However, regarding the patient examinations, there was not any massive hematoma or bruising at the right brow, which partially questions the paresis due to massive hematoma. Usually, SO paresis secondary to trochlear trauma recovers spontaneously after a few months. If the disorder remains stable after a follow‐up period of several months, surgical procedures including inferior oblique muscle weakening plus additional vertical and horizontal rectus surgery as needed, will help.^13^ Our patient's right eye extorsion and diplopia resolved spontaneously after 3 months of follow‐up.

## AUTHOR CONTRIBUTIONS

**Farid Shekarchian:** Conceptualization; data curation; writing – review and editing. **Mehrdad Motamed Shariati:** Supervision; writing – original draft; writing – review and editing. **Mitra Karimi Amir Abadi:** Data curation; visualization; writing – original draft; writing – review and editing.

## FUNDING INFORMATION

The authors received no funding.

## CONFLICT OF INTEREST STATEMENT

The authors declare that they have no conflicts of interest.

## CONSENT

Written informed consent was obtained from the patient to publish this report in accordance with the journal's patient consent policy.

## Data Availability

The data that support the findings of this study are available on request from the corresponding author. The data are not publicly available due to privacy or ethical restrictions.
